# Unilaterally extrapedicular versus transpedicular kyphoplasty in treating osteoporotic lumbar fractures: a randomized controlled study

**DOI:** 10.1186/s13018-023-04267-6

**Published:** 2023-10-26

**Authors:** Hao Hong, Jun Li, Haoyang Ding, Yi Deng, Zhongliang Deng, Qilong Jiang

**Affiliations:** 1 Department of Orthopaedic Surgery, Second Affiliated Hospital of Chongqing Medical University, Chongqing, China; 2 Department of Spinal Surgery, Chongqing Orthopedic Hospital of Traditional Chinese Medicine, Chongqing, China; 3 Department of Orthopaedic Surgery, Chongqing Orthopedic Hospital of Traditional Chinese Medicine, Chongqing, China

**Keywords:** Kyphoplasty, Extrapedicular, Osteoporotic vertebral fracture, Osteoporosis, Unipedicular

## Abstract

**Background:**

The unilaterally extrapedicular approach is adopted increasingly to perform balloon kyphoplasty in treating osteoporotic lumbar fractures, which is intended to improve radiological and clinical efficacy. We compared the efficacy and safety of this method with a unilaterally transpedicular approach.

**Methods:**

We conducted a single-center, randomized controlled trial enrolling participants with a one-level osteoporotic lumbar fracture in less than 1 month. Patients were randomly assigned to undergo kyphoplasty via either a unilaterally extrapedicular approach (treatment group) or a unilaterally transpedicular approach (control group). The primary outcome was the difference in change from baseline to 1 month in visual analog scale (VAS) scores between the two groups. Secondary outcome measures included vertebral height ratio, operation time, fluoroscopic times, hemoglobin loss, and cement leakage between groups. Data were analyzed by intention to treat principle.

**Results:**

A total of 80 participants were assigned to the treatment group (*n* = 40) and control group (*n* = 40), with three and two patients lost to follow-up during 12 months in the two groups, respectively. At 1 month postoperatively, the treatment group showed a greater reduction in VAS score from baseline, compared with the control group (mean difference between groups = 0.63, 95%CI 0.19–1.06). There were no significant between-group differences in restoration in anterior, middle, and posterior vertebral body (*P* > 0.05). No significant differences were found in the rate of cement leakage and perioperative hemoglobin loss (*P* > 0.05).

**Conclusion:**

Compared with balloon kyphoplasty via the unilaterally transpedicular approach in treating lumbar OVCFs, the unilaterally extrapedicular approach appears to be promising in achieving effective pain relief, adequate cement infusion, short operation time, less fluoroscopy exposure, and comparable risk of cement leakage and vessel injury. It is an alternative approach for lumbar OVCFs treated with kyphoplasty.

## Introduction

Osteoporotic vertebral compression fractures (OVCFs) have become one of the most common fragility fractures in the elderly population [1]. Symptomatic OVCFs can result in substantial pain and eventuate in decreased quality of life [2]. Percutaneous cement augmentation procedure, consisting of vertebroplasty and kyphoplasty, has exhibited superiority in pain relief over conservative management [3]. The balloon kyphoplasty is more frequently performed due to the advantage of vertebral height restoration and a lower rate of cement leakage [4, 5]. For osteoporotic thoracic fractures, the approach to injecting polymethylmethacrylate (PMMA) has evolved from bilaterally transpedicular, to unilaterally transpedicular, and further to unilaterally extrapedicular route [6–8]. The unilaterally extrapedicular approach has proven superiority in short operation time, symmetrical cement distribution, and a lower rate of cement leakage [9–11]. In recent years, several studies have reported the application of a unilaterally extrapedicular approach in treating lumbar OVCFs [12–14]. However, there are few prospective studies on the comparison between unilaterally extrapedicular versus unilaterally transpedicular approaches. Thus, we conducted the present randomized controlled study, aiming to clarify the clinical efficacy and safety of this emerging approach to carrying out balloon kyphoplasty for lumbar OVCFs. We hypothesized that the unilaterally extrapedicular approach would yield better clinical efficacy with a comparable complication rate compared to the unilaterally transpedicular approach.

## Methods

This randomized controlled trial was approved by the Institutional Review Board of the Chongqing Orthopedic Hospital of Traditional Chinese Medicine. Written informed consent was obtained from all participants involved in the present study.

### Participants

**Fig. 1:**
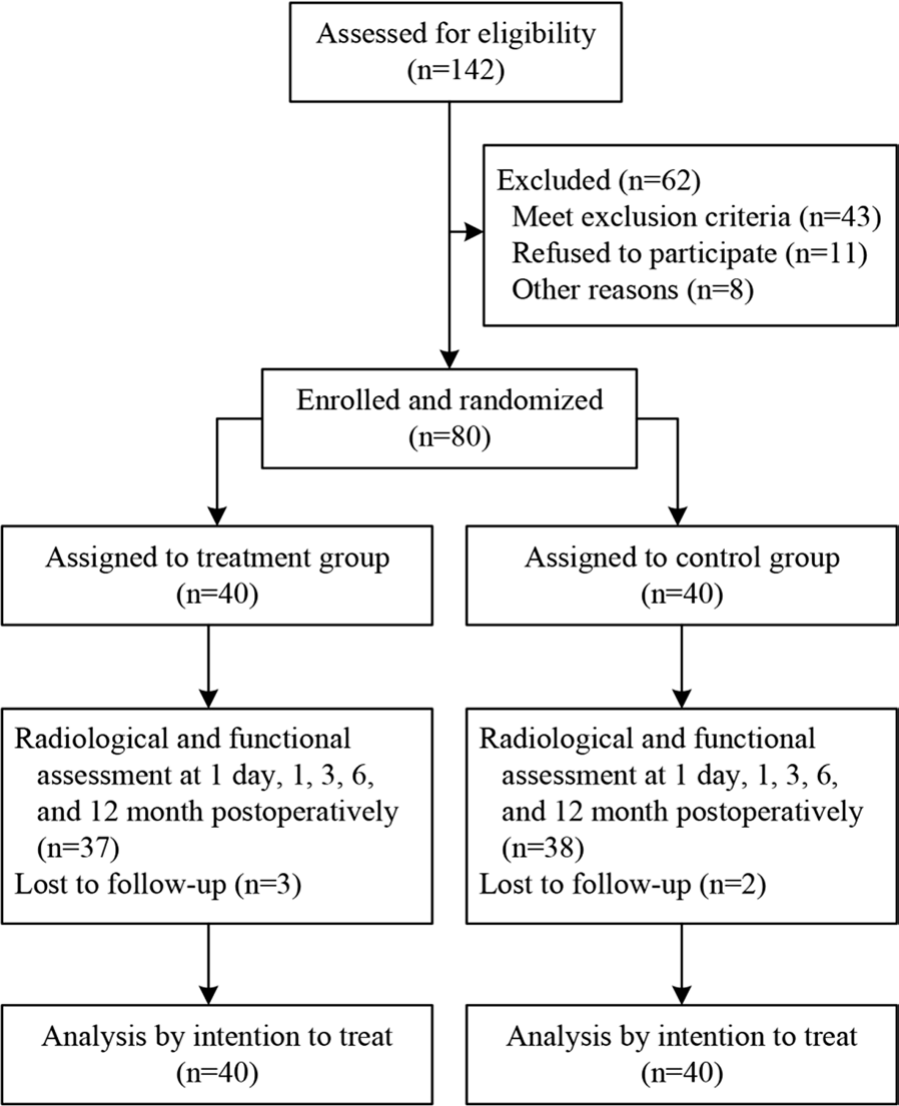
Trial profile

From May 2020 to May 2022 in a single institute, all patients with back pain within 1 month were screened for eligibility (Fig. 1). Magnetic resonance imaging (MRI) and dual-energy X-ray absorptiometry (DEXA) scans were performed to identify OVCFs. The presence of vertebral marrow edema on MRI imaging indicates an acute fracture. Only patients with one-level acute lumbar OVCF were included in this trial. The severity of osteoporosis was assessed by bone mineral density T score. Exclusion criteria were patients (1) with myeloma, osteoblastic metastases, or osteolytic metastatic tumors, (2) with a back pain score of fewer than 4 points on a 0–10 visual analog scale (VAS) scores, (3) with canal narrowing or radicular pain, (4) younger than 50 years old.

### Interventions

Based on computer-generated random numbers, we randomly assigned enrolled patients to undergo unilateral balloon kyphoplasty either by an extrapedicular approach (treatment group) or a transpedicular approach (control group). Individuals were blinded to the assigned group throughout their hospital stay and follow-up. All surgical interventions were performed by the same surgeon aware of patient information and corresponding assignment.

**Fig. 2:**
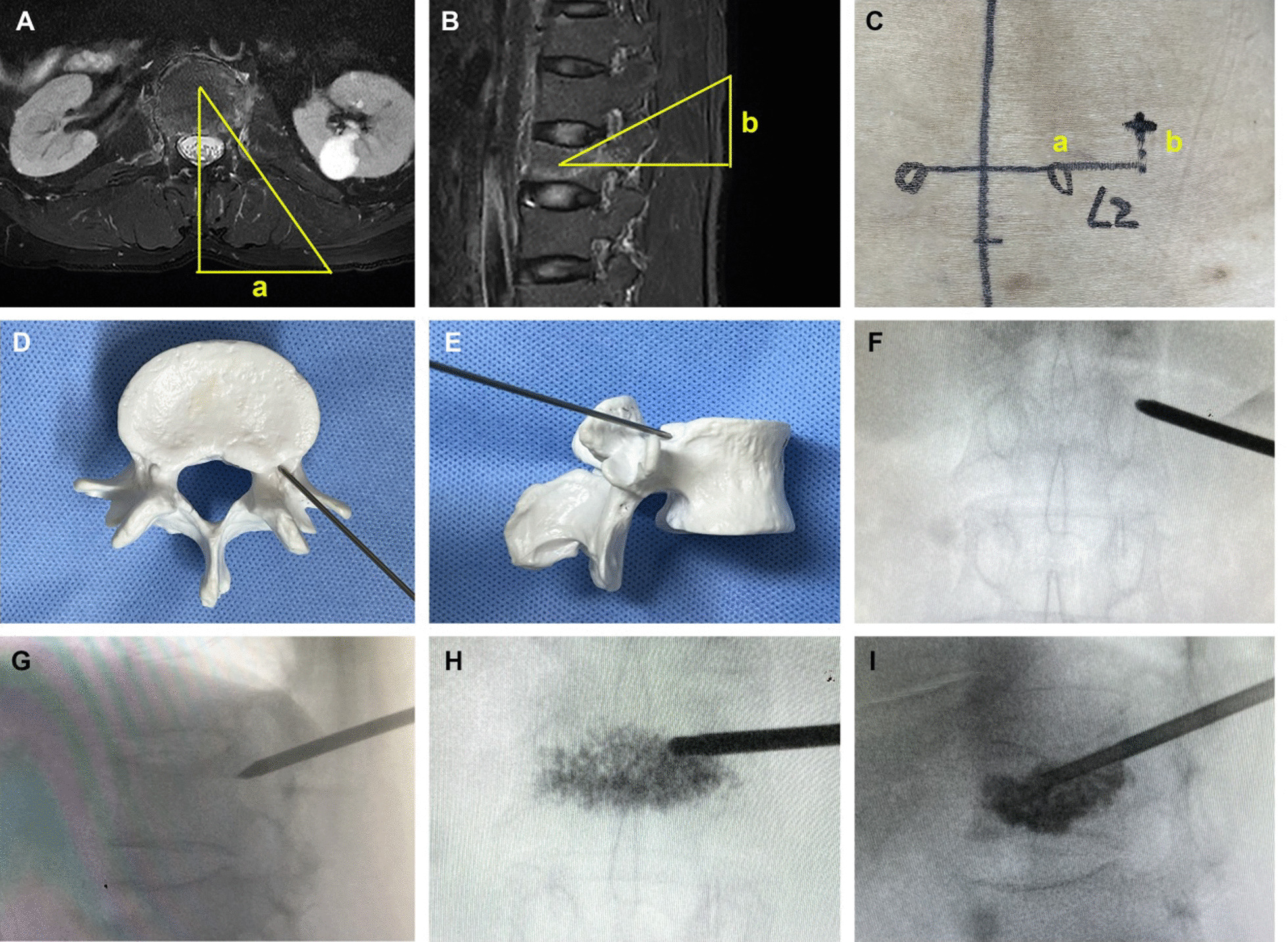
Kyphoplasty by unilaterally extrapedicular approach. **A** On the axial MRI film, a indicates the distance between the spinous process and the puncture site. **B** On the sagittal MRI film, b indicates the distance between the puncture site and the line crossing pedicles. **C** The puncture site on skin based on preoperative MRI measurements. **D**, **E** the entry site is located on the superolateral periosteum relative to the pedicle. **F**, **G**, **H**, **I** the surgical procedure of kyphoplasty by the unilaterally extrapedicular approach

For the treatment group, the highlight of the extrapedicular approach was that the insertion site was located on the superolateral periosteum relative to the pedicle, which could provide a higher puncture angle on both the sagittal and horizontal plane (Fig. 2). The detailed procedures were as follows. Based on the preoperative MRI imaging, the insertion site and angle on the skin could be measured and noted (Fig. 2A, B). During the surgical procedure, patients were placed in the prone position on a radiolucent table. A C-arm or G-arm was placed on the contrary side to carry out intraoperative fluoroscopy. One or two metallic markers were used to identify the pedicle of the target vertebrae. Then, the puncture site and angle on the skin were marked with the usage of pre-measured data (Fig. 2C). After the infiltration with 1% lidocaine or 0.25% bupivacaine, a needle was inserted in the predesigned direction. It was essential to confirm the needle reached the correct periosteum under fluoroscopic guidance (Fig. 2D–G). After that, an inflatable bone tamp (IBT) was inserted inside the middle third of the vertebral body on both anterior–posterior and lateral images. The balloon was then inflated and the cement was injected slowly until satisfactory infiltration was achieved (Fig. 2H, I). Generally, the volume of cement was controlled up to 6 mL for each vertebra. The injection was also stopped if cement leakage occurred (e.g., extraosseous and spinal leakage).

For the control group, all patients received unilaterally transpedicular balloon kyphoplasty. We did not allow conversion to bipedicular kyphoplasty even if the unilateral injection failed to achieve adequate instillation of cement, which could improve the comparability between groups. The surgical procedures were done according to standard practices of introducer instruments.

Following the surgery, all patients stayed in the hospital for 1–2 days and received ancillary care (e.g., osteoporosis drugs, back braces, analgesics, and physiotherapy) when necessary. A subsequent vertebral fracture was treated as the initial assignment.

### Outcome assessment

Radiological assessment was done by a blinded radiologist while functional assessment was done by another blinded assessor. Baseline data included gender, age, body mass index (BMI), bone mineral density (BMD), the time from injury to surgery, affected lumbar level, vertebral body height ratio, and VAS scores. If injury time was uncertain, the onset of back pain was recorded. Vertebral height was presented as anterior, middle, and posterior vertebral body height ratios, which were measured on lateral radiographs. Assessment during operation involved operation time, fluoroscopic times, injected cement volume, and occurrence of cement leakage. The cement leakage was defined as leakage in any direction extraosseously. The hidden blood loss was evaluated as the reduction of perioperative hemoglobin value. Following the surgery, all patients underwent both functional (VAS scores) and radiological (anterior–posterior and lateral images) assessments at postoperative 1 day, 1, 3, 6, and 12 months of follow-up. If a participant failed to visit assessors in our hospital, telephone contact was carried out. Radiographs could be performed at local hospitals and sent to us via WeChat or Email. The intention to treat principle was used for missing data.

### Statistical analysis

The primary endpoint was the difference in change from baseline to 1 month in VAS scores between the treatment and control groups. With the usage of PASS software, we calculated that per group would include at least 34 participants (90% power, 5% type 1 error rate, 1.24 standard deviation, a change of 1 point on VAS score). We used SAS software version 9.4 to conduct the statistical data analysis. Mixed effects modeling was used to analyze repeated measures (VAS scores and vertebral height ratio). The Chi-square test or Fisher’s exact test was used for categorical variables. Continuous data were tested for normality using the Shapiro–Wilk test. For data matching normal distribution, a two-sample t test was used to detect statistical differences between groups. The Mann–Whitney U test was used for continuous variables with non-normal distribution. The differences between groups were presented with 95% confidence intervals. A *P* value of < 0.05 was considered significant. We produced statistical figures with the use of GraphPad Prism software version 9.5.1.

## Results

A total of 142 participants were assessed for eligibility. Finally, 80 patients were randomly allocated to the treatment group (*n* = 40) or control group (*n* = 40). At baseline, the characteristics between the two groups were similar (Table 1). From randomization to 12-month follow-up, 37 patients in the treatment group and 38 patients in the control group had complete data. A total of six patients encountered a second OVCF (three in each group) and received the same protocol as the initial assignment. All 80 participants were taken into the intention to treat analysis.

**Table 1:** Baseline characteristics of participants

	Treatment group (*n* = 40)	Control group (*n* = 40)
*Gender*		
Male	3	6
Female	37	34
Mean (SD) age	73.64 (7.15)	72.84 (6.94)
Mean (SD) body mass index	23.74 (3.73)	23.41 (3.52)
Mean (SD) bone mineral density	− 3.21 (0.32)	− 3.25 (0.33)
Median (IQR) days from injury to kyphoplasty	4.5 (3.5)	4.5 (3.5)
*Lumbar level*		
L1	15	14
L2	13	12
L3	8	9
L4	3	3
L5	1	2

**Fig. 3:**
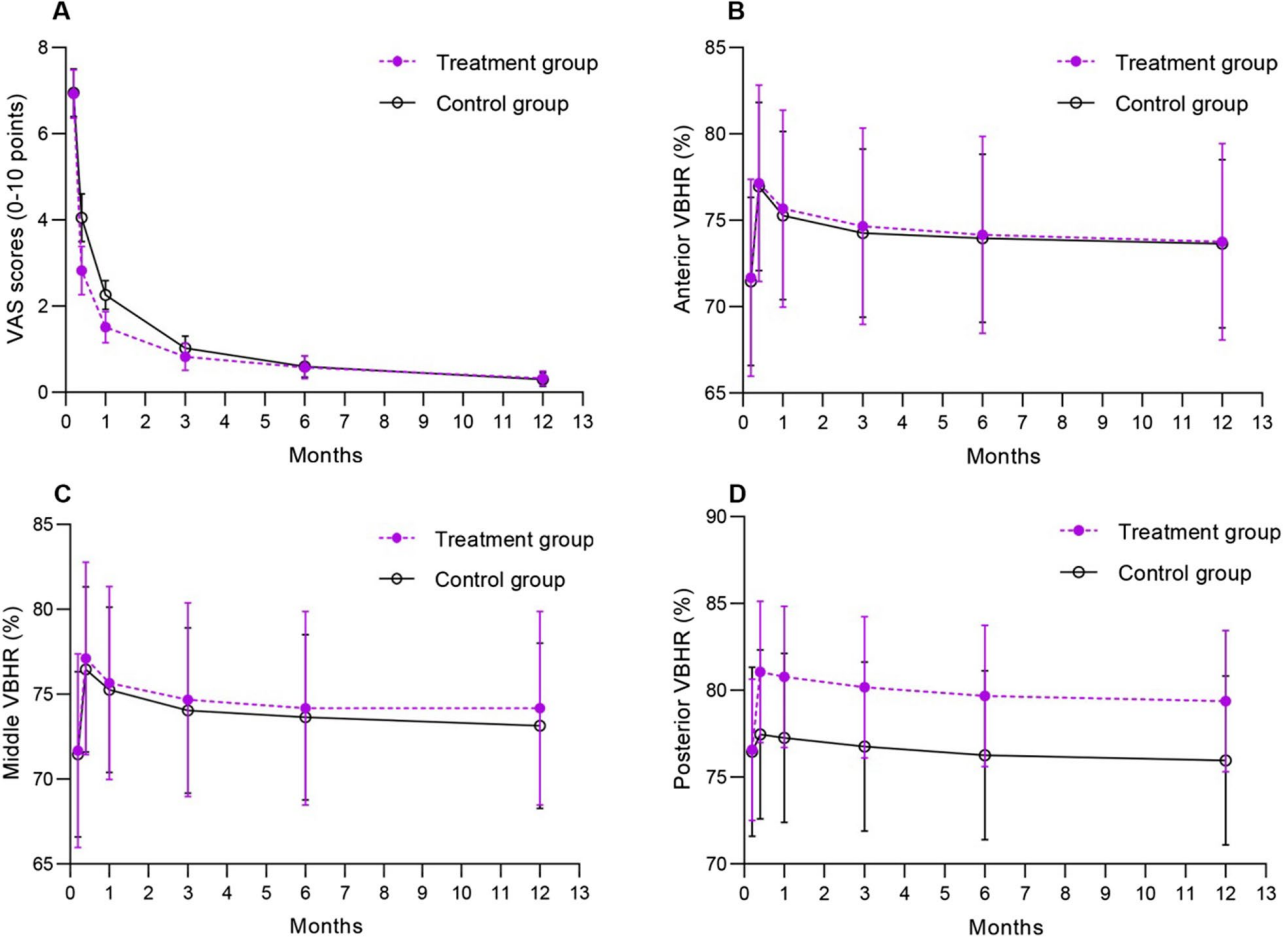
Functional and radiological assessment during preoperation and 12-month follow-up (1 day, 1, 3, 6, and 12 months postoperatively). Means with 95% confidence intervals were presented. The first measurement in each figure indicates baseline data. **A** visual analog scale (VAS) scores. **B, C, D** anterior, middle, and posterior vertebral body height ratios (VBHR), indicating height loss of vertebral body, were measured on lateral radiographs

For the primary outcome, a significant interaction was detected between the intervention effect and follow-up time. The treatment group showed a greater reduction in the VAS score from baseline to 1 month, compared with the control group (adjusted MD = 0.63, 95%CI 0.19–1.06). At 1 day postoperatively, there were also significant differences in the VAS score reduction between groups (adjusted MD = 1.13, 95%CI 1.02–1.23). At 3, 6, and 12 months, no between-group differences were found in the VAS score reduction (Fig. 3).

In the radiological assessment, no interaction was found significant between treatment and follow-up time (Fig. 3). There were no significant differences in anterior, middle, and posterior vertebral body height ratios between groups (all *P* > 0.05).

The treatment group exhibited shorter operation time of 2.85 min than the control group (95%CI 1.54–4.16, *P* < 0.001). More cement could be injected into the target vertebral body in the treatment group (MD = 5 ml, 95%CI 0.98–0.92, *P* < 0.001). Six and nine patients encountered cement leakage in the treatment group and control group, respectively. No significant associations were found between groups and the incidence of cement leakage (relative risk = 0.67, 95%CI 0.26–1.70, *P* > 0.05). There were no significant between-groups differences in hemoglobin loss (Table 2).

**Table 2:** Assessment during operation

	Treatment group (*n* = 40)	Control group (*n* = 40)	Mean difference or relative risk	*P* value
Operative duration (min)*	34.20 (33.2 to 35.12)	37.05 (36.09 to 38.01)	− 2.85 (− 4.16 to − 1.54)	< 0.001
Fluoroscopic times*	16.85 (16.12 to 17.58)	16.60 (15.84 to 17.36)	0.25 (− 0.79 to 1.29)	0.634
Hemoglobin loss (g/L)*	9.37 (8.10 to 10.63)	9.30 (8.15 to 10.45)	0.06 (− 1.62 to 1.75)	0.941
Cement volume (ml)*	4.83 (4.47 to 5.18)	3.38 (3.05 to 3.70)	1.45 (0.98 to 1.92)	< 0.001
Cement leakage^#^	0.15 (0.06 to 0.30)	0.23 (0.11 to 0.39)	0.67 (0.26 to 1.70)	0.40

*Indicates the data presented with mean (95% CI) and mean difference (95% CI)

^#^Indicates the data presented with rate (95% CI) and relative risk (95% CI)

## Discussion

The most important finding of the present randomized controlled trial was that in patients with lumbar OVCFs, balloon kyphoplasty by unilaterally extrapedicular approach could yield effective pain relief, adequate cement infusion, short operation time, less fluoroscopy exposure, and comparable risk of cement leakage and vessel injury, compared with the procedure by the unilaterally transpedicular approach.

The unilaterally extrapedicular approach for kyphoplasty has prevailed over the past years in treating thoracic OVCFs [6]. A myriad of studies has stated that both extrapedicular and transpedicular approaches are equally effective and safe in relieving pain for patients undergoing thoracic kyphoplasty [15–17]. In recent years, some attempts were made to use the unilaterally extrapedicular approach to perform kyphoplasty in treating lumbar OVCFs. Zhu et al. [14] retrospectively analyzed 76 lumbar OVCFs treated with the percutaneous kyphoplasty by either a bilateral transpedicular approach or unilateral extrapedicular approach. The results found that there were no between-group significant differences in both VAS and Oswestry Disability Index scores throughout a mean follow-up of 16.6 months. However, to our best knowledge, there were still no randomized controlled trials to compare the extrapedicular and transpedicular approach in performing unilateral kyphoplasty for lumbar OVCFs. In the current study, the treatment group showed a significantly greater reduction in the VAS score than the control group within 1 month of follow-up. However, the differences were no longer significant at 3, 6, and 12 months postoperatively. An explanation is that the extrapedicular approach could more easily access the center of the fractured vertebra and provide more symmetric cement filling than the transpedicular approach. This was possibly attributed to greater pain relief in the extrapedicular approach group. The finding of this study was consistent with prior evidence that symmetric cement infusion is more effective than asymmetric filling in pain relief [18]. Although there is still a lack of randomized controlled trials comparing the unilaterally extrapedicular approach with the bilaterally transpedicular approach in lumbar kyphoplasty, based on clinical practices; however, we believe that the two approaches are comparable to achieve symmetric cement infusion and pain relief. Another possible benefit of the extrapedicular approach in pain relief is the avoided injury of facet joint and capsule, which is reported to be associated with residual pain after transpedicular kyphoplasty [19].

The cement infiltration by the unilaterally extrapedicular approach brought out additional advantages over the transpedicular approach. In this study, compared to the control group, more cement was injected inside the target vertebral body in the treatment group. We deduce that the achievement of satisfactory cement infusion is highly associated with an advantageous puncture site and angle. In previous studies, several extrapedicular approaches have been proposed. Ryu et al. [20] proposed a far-lateral extrapedicular approach, by which the needle penetrated the transverse process with a puncture angle of 45 to 50 degrees on the horizontal plane. Cho et al. [21] introduced a personalized approach measured before surgery, in which the puncture was advanced superiorly to the upper edge of the transverse process. Wang et al. [22] suggested performing a puncture through Kambin’s triangle. The entry point was located at the junction between the pedicle and vertebral body. In the present study, we proposed a modified extrapedicular approach. Specifically, the insertion site and angle were based on the anatomical landmarks on radiographs preoperatively. In the surgical procedure, the needle tip reached the bottom of Kambin’s triangle at a puncture angle of 45 degrees from the sagittal plane. The puncture trajectory could easily access the center of the fractured vertebra. A single balloon was sufficient to produce symmetrical expansion and achievement of adequate return of kyphotic angle. In this trial, the majority of the unilaterally extrapedicular injection has produced similar cement infusion with traditional bilaterally transpedicular kyphoplasty. Moreover, although a mean of 4.83 ml cement was injected into a fractured lumbar vertebra, the risk of cement leakage did not differ between the extrapedicular and transpedicular approaches. The overall rate of cement leakage accounted for 15.0% (6/40), which coincided with prior studies [23, 24].

The most common concerns about the extrapedicular approach are puncture-related injuries involving segmental artery injury and nerve root lesions. Heo et al. [25] performed a spinal computed tomographic angiography and found that the segmental arteries predominantly passed below the midline of the pedicle. By the extrapedicular approach in the present study, the insertion site was located superior-laterally to the pedicle. The needle tip was controlled to slip on the periosteum and pass through the posterolateral lamina. Theoretically, the injury of segmental arteries could be avoided by this method. We calculated perioperative hemoglobin reduction to assess hidden blood loss. Between the extrapedicular and transpedicular approaches, no significant differences were found in perioperative hemoglobin loss. However, the definitive identification of retroperitoneal hematoma could be confirmed by an abdominal CT scan, especially for patients with hypovolemic shock and progressive hemoglobin loss after an extrapedicular kyphoplasty [8, 25]. Concerning the nerve root injury, which could be identified easily by the occurrence of radicular pain, both extrapedicular and transpedicular approaches in our study were proven to be equally safe in performing lumbar kyphoplasty.

As all kyphoplasty procedures in this study were carried out by an experienced surgeon, no significant clinical differences have been shown between the two approaches. Actually, the learning curve of the extrapedicular approach is much longer than that of the transpedicular approach for inexperienced practitioners. It will take more time and fluoroscopy to reach the optimal insertion point on the periosteum and verify the occurrence of malposition and cement leakage. As the experience begins to accumulate, the procedure of kyphoplasty by extrapedicular approach could be performed with much shorter time and less fluoroscopy exposure.

This study had several limitations. First, the surgeon involved in the surgical procedure was unblinded to participants, which may lead to performance bias. Second, there was no assessment of the quality of life (e.g., Roland-Morris disability questionnaire scores, short-form 36 physical component summary score), which may reach insufficient conclusions. Third, this study compared only unilaterally extrapedicular and transpedicular approaches. The clinical significance would be elevated if the bilaterally transpedicular approach could be taken into comparison.

## Conclusions

The unilaterally extrapedicular approach appears to be a promising method to perform kyphoplasty for patients with lumbar OVCFs. It combines the advantages of unilaterally and bilaterally transpedicular approaches, with effective pain relief, adequate cement infusion, short operation time, less fluoroscopy exposure, and comparable risk of cement leakage and vessel injury. It is a viable option for carefully selected patients with lumbar OVCFs.

## Data Availability

The datasets used and/or analyzed during the current study are available from the corresponding author on reasonable request.

## Abbreviations

OVCFs: Osteoporotic vertebral compression fractures
VAS: Visual analog scale
PMMA: Polymethylmethacrylate
MRI: Magnetic resonance imaging
DEXA: Dual-energy X-ray absorptiometry
IBT: Inflatable bone tamp
BMI: Body mass index
BMD: Bone mineral density
VBHR: Vertebral body height ratios
